# Reemergence of Yellow Fever, Magdalena Valley, Colombia, 2024–2025

**DOI:** 10.3201/eid3112.251209

**Published:** 2025-12

**Authors:** Jerson Andrés Cuéllar-Sáenz, Alfonso J. Rodríguez-Morales, Álvaro A. Faccini-Martínez

**Affiliations:** Universidad Nacional de Colombia, Bogotá, Colombia (J.A. Cuéllar-Sáenz); Pontificia Universidad Javeriana, Bogotá (J.A. Cuéllar-Sáenz); Faculty of Health Sciences, Universidad Científica del Sur, Lima, Peru (A.J. Rodríguez-Morales); Grupo de Investigación Biomedicina, Faculty of Medicine, Fundación Universitaria Autónoma de las Américas-Institución Universitaria Visión de las Américas, Pereira, Colombia (A.J. Rodríguez-Morales); Hospital Militar Central, Bogotá (Á.A. Faccini-Martínez); Universidad Militar Nueva Granada, Bogotá (Á.A. Faccini-Martínez)

**Keywords:** yellow fever, viruses, vector-borne infections, Colombia, One Health

## Abstract

Yellow fever, a zoonotic arboviral disease, has reemerged in Colombia, triggering a major outbreak in the country. During 2024 through mid-2025, a total of 132 human cases and 68 infections in nonhuman primates were confirmed, primarily in the department of Tolima, historically considered a low-risk area. We analyzed the historical and current epidemiology of yellow fever in Colombia, highlighting ecologic, social, and surveillance factors that contributed to the outbreak. Low vaccination coverage, insufficient epizootic and entomological surveillance, deforestation, habitat fragmentation, and limited application of One Health approaches have all exacerbated the situation. The high mortality rate of nonhuman primate species indicated a more profound ecologic crisis. Immediate, comprehensive measures, including mass vaccination, genomic surveillance, and integrated One Health frameworks, are urgently needed. Colombia’s experience underscores the need to reevaluate risk stratification and preparedness strategies across the Americas to prevent future yellow fever outbreaks in previously unaffected regions.

Yellow fever is an acute viral disease caused by an arbovirus (*Orthoflavivirus flavi*) of the genus *Flavivirus*. It is transmitted by vectors, specifically by hematophagous mosquitoes of the genera *Haemagogus* and *Sabethes* for the sylvatic cycle and *Aedes aegypti* for the urban cycle (*1*). As of 2025, there is no specific treatment for the disease; however, vaccination is an effective preventive measure (*2*).

## Yellow Fever in the 19th and 20th Century

Yellow fever first appeared in Colombia during the 16th and 17th Centuries. The first documented epidemic cases occurred in Cartagena and Santa Marta in 1729; later cases were identified in soldiers arriving on the Atlantic coast from Spain (*3*). During 1830–1900, cases were reported in the Magdalena and Catatumbo Valleys, characterized by notable clinical and epidemiologic descriptions; the populations most affected were farmers, rainforest workers, soldiers, and railway workers. An outbreak in 1830 resulted in 1,800 deaths out of 4,000 inhabitants in Ambalema, Tolima Department (*4*). At the beginning of the 20th Century, outbreaks were reported in Soto’s Valley (now in the Santander department) during 1910–1912 and in 1923 (Table 1) (*4*–*7*). Those cases were characterized as a sylvatic outbreak, with the presence of *Haemagogus* spp. mosquitoes (*5*). Until the 1930s, yellow fever cases were associated with *Ae*. *aegypti* mosquitoes as the vector; the paradigm shifted with Franco’s work in Muzo in 1907 (*8*) and Soper’s work in 1937 (*3*), which revealed that yellow fever infections in Colombia were part of the sylvatic cycle. In 1929, the last urban cases of the disease, which involves human–vector–human transmission through the bite of the *Ae. aegypti* mosquito, were reported in Socorro, Santander (Figure 1) (*9*).

**Table 1:** Years of reported cases of yellow fever in Colombia, by department and municipality, during the 19th and early 20th Centuries noted in study of reemergence of yellow fever in Colombia, 2024–2025*

Department	Municipality	Years
Antioquia	Aquitania	1934–1956
	Caracolí	1934–1956
	Maceo	1934–1956
	Malena	1915
	Mutatá	1947
	Puerto Berrío	1934–1956
	Remedios	1934–1956
	San Carlos	1934–1956
	San Luis	1934–1956
	Yolombó	1934–1956
Atlántico	Barranquilla	1872, 1889, 1912
	Unespecified	1912
Bolívar	Cartagena	1872, 1885, 1912
	Mompox	1856, 1865, 1866
	Simití	1934–1956
	Tacamocho	1915
Boyacá	Puerto Boyacá	1934–1956
	Alto del Roble	1934–1956
	Borbur	1934–1956
	Briceño	1934–1956
	Campohermoso	1934–1956
	Cháneres	1934–1956
	Maripí	1934–1956
	Muzo	1906, 1907, 1916, 1923, 1924, 1934–1956
	Otanche	1934–1956
	Pauna	1934–1956
	San Rafael	1934–1956
Caldas	Buenavista	1934–1956
	Florencia	1934–1956
	La Dorada	1934–1956
	Manizales	1915, 1915
	Norcasia	1934–1956
	Samaná	1934–1956
	Victoria	1934–1956
Caquetá	San Vicente del Caguán	1934–1956
	Tres esquinas	1934–1956
Casanare	Nunchía	1934–1956
	Ten	1919–1923, 1934–1956
	Támara	1934–1956
Cesar	La Paz	1945
	Río de Oro	1934–1956
Cundinamarca	Anapoima	1900
	Cachipay	1934–1956
	Caparrapí	1934–1956
	Girardot	1865
	Guaduas	1857, 1879, 1885, 1880
	Jerusalén	1889
	La Mesa	1910, 1934–1956
	La Palma	1934–1956
	Medina	1934–1956
	Paime	1934–1956
	Peñalisa	1865
	Tocaima	1884
	Ubalá	1934–1956
	Yacopí	1934–1956
Huila	Aipe	1881
	Neiva	1881
La Guajira	Fonseca	1945
	San Juan de César	1945
Magdalena	Santa Marta	1887
Meta	Acacías	1934–1956
	Cumaral	1934–1956
	Guamal	1934–1956
	Puerto López	1934–1956
	Restrepo	1934–1956
	San Martín	1934–1956
	Villavicencio	1934–1936
Norte de Santander	Cáchira	1934–1956
	Cúcuta	1883, 1880, 1887, 1947
	El Carmen	1888, 1907
	Ocaña	1888, 1907
	Villa del Rosario	1883, 1886, 1900
	San Cayetano	1884, 1886, 1900
	Sardinata	1934–1956
	Tibú	1934–1956
Putumayo		
	Mocoa	1934–1956
	Puerto Leguízamo	1934–1956
Quindío	La Pradera	1934–1956
Santander	Barrancabermeja	1934–1956
	Bucaramanga	1910–1912, 1919, 1923
	Florián	1934–1956
	Floridablanca	1910–1912, 1929
	Girón	1910–1912, 1929
	Guadalupe	1900
	Jordán	1934–1956
	La Belleza	1934–1956
	Landázuri	1934–1956
	Lebrija	1934–1956
	Piedecuesta	1910–1912
	Puerto Wilches	1934–1956
	Rionegro	1910–1912
	San Joaquín	1900
	San Vicente de Chucurí	1929, 1934–1956
	Santa Helena	1934–1956
	Socorro	1929
	Vélez	1934–1956
Tolima	Ambalema	1830, 1858, 1881
	Armero	1934–1956
	Espinal	1870, 1871, 1879, 1880, 1881
	Honda	1830, 1857, 1872, 1879, 1884
	Mariquita	1934–1956
	Purificación	1884
Valle del Cauca	Buenaventura	1907, 1915, 1920
	Cali	1920
	La Unión	1881

*Sources: Gast Galvis (*4*), Gast Galvis (*5**)*, Boshell Manrique (*6*), Gast Galvis (*7*).

**Figure:**
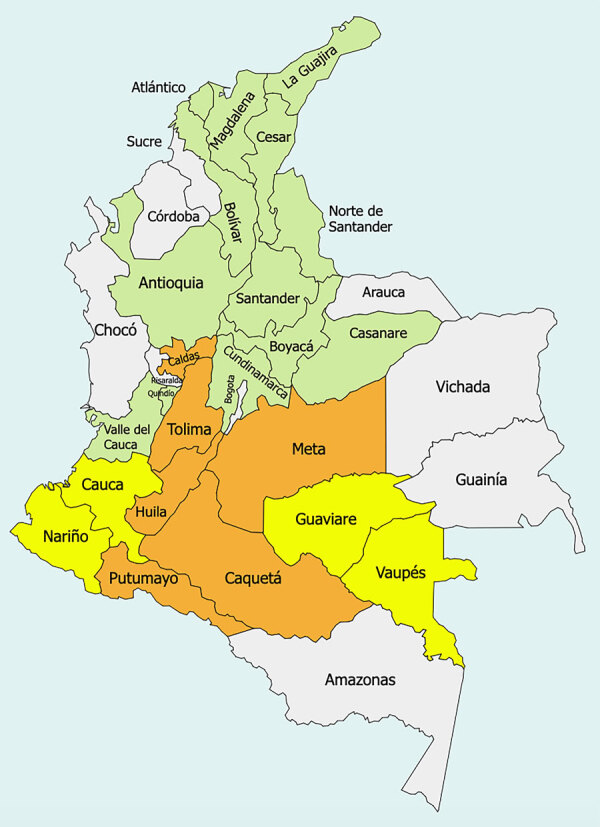
Departments of Colombia that have reported cases of yellow fever. Green indicates historical cases of yellow fever during the 19th and early 20th Centuries, yellow indicates cases during the current 2024–2025 outbreak, and orange indicates cases during both periods.

Gast Galvis (*5*) analyzed 38,275 liver samples collected during 1934–1956, identifying 594 positive cases in the Amazon region, the foothills of the Eastern plains, the Magdalena Valley, and the Catatumbo River basin. The cases were not clinically diagnosed before death. Limited information is available on cases collected during the remaining years of the 20th Century (Table 2) (*10*–*13*).

**Table 2:** Numbers of reported cases of yellow fever in Colombia during 1956–1999 noted in study of reemergence of yellow fever in Colombia, 2024–2025*

Years	Cases
1956–1962	ND
1963	10
1964	10
1965	2
1966	3
1967	5
1968	11
1969	7
1970	7
1971	9
1972	3
1973	16
1974	36
1975	12
1976	22
1977	9
1978	105
1979	51
1980	11
1981	7
1982	2
1983	1
1984	16
1985	4
1986–1999	5

*Sources: Segura et al. (10), Pan American Health Organization (*11**–**13*). ND, no data.

## Yellow Fever in the 21st Century

During 2000–2023, Colombia reported 216 confirmed cases of yellow fever in the departments of Norte de Santander, Magdalena, Guajira, Vichada, Meta, Caquetá, Putumayo, Guaviare, Chocó, Amazonas, Guainía, Vaupés, and Santander (Table 3) (*14*,*15*). An outbreak occurred in the Catatumbo region of the Norte de Santander department and the Sierra Nevada de Santa Marta region (comprising the César, La Guajira, and Magdalena departments); of 216 cases, 102 (46%) occurred in 2003 and 31(14%) in 2004 (*9*,*16*). The mortality rate was 44% in 2003 and 36% in 2004. Both Colombia and Venezuela used vaccination to control the outbreak on their shared border (*17*).

**Table 3:** Numbers of reported cases of yellow fever in Colombia during 2000–2023 noted in study of reemergence of yellow fever in Colombia, 2024–2025*

Year	Cases
2000	5
2001	8
2002	20
2003	102
2004	31
2005	20
2006	5
2007	7
2008	3
2009	5
2010	0
2011	0
2012	0
2013	1
2014	0
2015	0
2016	7
2017	0
2018	1
2019	0
2020	0
2021	0
2022	0
2023	2
Total cases	216

*Source: Instituto Nacional de Salud de Colombia (*14**,**15*).

The Tolima department (within the Magdalena Valley) had no historical reports during 2000–2024; therefore, it was not considered a high-risk region for yellow fever (*2*). Nevertheless, 7 cases were confirmed in October 2024 in neighboring villages in the southwestern part of the Bosque de Galilea Regional Natural Park, which includes areas of 4 municipalities in Tolima (Cunday, Prado, Villarrica, and Purificación) on the border with the Huila and Cundinamarca departments (*18*). The Tolima department was classified as low stratification risk in accordance with the national plan for the prevention and control of yellow fever in Colombia during 2017–2022 (*2*). 

As of September 18, 2025, a total of 132 confirmed cases of yellow fever had been reported during 2024–2025 in Colombia, distributed across 10 departments: Tolima (n = 112 cases), Putumayo (n = 8), Meta (n = 3), Caquetá (n = 2), Nariño (n = 2), Vaupés (n = 1), Caldas (n = 1), Cauca (n = 1), Huila (n = 1), and Guaviare (n = 1) (Table 4; Figure) (*19*). Those cases represent an estimated incidence of 2.51 cases/1 million population for Colombia (0.25 cases/100,000 population), with higher values for the most affected municipalities (e.g., 382.6 cases/100,000 population in Villarica, Tolima) (Table 4); case-fatality rate was 39.4%, and cumulative mortality rate was 0.99 deaths/1,000,000 population (*19*). Most cases were observed among persons residing in rural areas who have not received the vaccination; median age was 45 years, and sex distribution was 27 women and 105 men (*19*). In contrast, the virus has been confirmed in 68 nonhuman primates (NHPs) in 4 departments: 51 cases in Tolima, 8 in Huila, 8 in Putumayo, and 1 in Meta (*19*). To contain this outbreak, the Ministerio de Salud y Protección Social of Colombia, by Resolution 691 of 2025, declared a health emergency throughout the country and adopted measures for infection prevention and control (*20*). The measures included declaration of a public health emergency, mass vaccination campaigns, intensified vector control, strengthened epidemiologic and epizootic surveillance, and community risk communication, although coverage remained below optimal levels and thus insufficient to fully contain transmission.

**Table 4:** Reported cases of yellow fever in Colombia, by department and municipality, noted in study of reemergence of yellow fever in Colombia,

2024–2025*Location	No. cases	No. cases/100,000 population
Tolima	112	
Villarica	20	382.6
Cunday	19	215.9
Prado	17	192.8
Ataco	26	129.3
Dolores	4	46.3
Rioblanco	7	29.9
Purificación	7	29.1
Valle de San Juan	1	18.0
Chaparral	8	14.6
Palocabildo	1	10.2
Espinal	1	1.3
Ibagué	1	0.2
Putumayo	8	
Villagarzón	2	7.4
Orito	4	5.3
San Miguel	1	4.6
Valle del Gamuez	1	1.9
Caquetá	2	
El Doncello	1	2.4
Cartagena del Chairá	1	1.2
Nariño	2	
Ipiales	2	1.6
Meta	3	
La Macarena	1	3.4
San Martín	1	3.1
Granada	1	1.3
Vaupés	1	
Mitú	1	2.9
Caldas	1	
Neira	1	4.6
Cauca	1	
Piamonte	1	10.2
Huila	1	
Campoalegre	1	3.0
Guaviare	1	
San José del Guaviare	1	1.8
Total cases	132	

*Source: Instituto Nacional de Salud de Colombia (*19*). Total no. cases is as of September 18, 2025.

## Perspectives

For decades, yellow fever has been a public health threat in the Americas because of its emergence and reemergence (*21*). At the same time, it has been recognized that Colombia has an underreported rate, which makes analyzing and controlling cases difficult (*10*). For some time, the importance of improving studies and vigilance regarding vectors (*Aedes* spp., *Haemagogus* spp., and *Sabethes* spp. mosquitoes) and nonhuman primate reservoirs (*Alouatta* spp., *Saimiri* spp., *Ateles* spp., *Aotus* spp., *Callithrix* spp., *Brachyteles* spp., *Callicebus* spp., *Leontopithecus* spp., and *Sapajus* spp. monkeys) has been highlighted, as well as the ecologic characteristics of regions with medium or high risk in Colombia to strengthen vigilance systems (*22*,*23*). Unfortunately, epizootic and vector surveillance are only considered after outbreaks in some cases. Therefore, strong surveillance has been implemented since the ongoing outbreak (*24*).

NHPs are considered natural sentinel species for the early detection of yellow fever epidemics. NHPs have a clinical course and mortality rate similar to those experienced by humans, and they have a substantial epidemiologic nexus in areas where outbreaks occur. A One Health perspective would consider NHPs’ conservation, the ecosystems in which they coexist with humans, and the effects of climate change on vector distribution (*25*). In Colombia, the distribution of *Ae. aegypti* mosquitoes in densely populated urban centers, coupled with the presence of *Haemagogus* and *Sabethes* mosquito species in sylvatic corridors, creates overlapping transmission zones that vary by ecosystem. At the same time, NHPs such as howler monkeys (*Alouatta* spp.), spider monkeys (*Ateles* spp.), and squirrel monkeys (*Saimiri* spp.) act as reservoirs and sentinel hosts; deaths in those animal populations often precede human cases. Those ecologic dynamics are especially evident in departments such as Tolima, Putumayo, and Caquetá, where expanding agricultural frontiers, deforestation, and human settlement bring humans into closer contact with vectors and primate reservoirs, underscoring how ecosystem-specific interactions directly shape the distribution and risk for yellow fever outbreaks across the country. Such outbreaks have been associated with relevant declines in NHP populations, which endanger mammalian biodiversity and influences the epidemiologic behavior of yellow fever (*26*). 

In 2025, Brazil, Colombia, Peru, and Bolivia reported cases or outbreaks outside the Amazon region, including cases in other ecologic corridors or ecosystems (*27*). That information is particularly important in the context of the correlation between deforestation, habitat fragmentation, and the destruction of NHPs’ habitats because those factors increase contact between humans and NHPs and affect the ecology of emerging infectious diseases (*28*). Approximately 38 species of NHPs are found in Colombia, and 10 of these species are endemic; their populations have declined from deforestation for illegal logging, expansion of the agricultural frontier, use for illicit crops, and mineral extraction, among other factors (*2*,*29*,*30*). At the same time, Colombia has recognized the correlation between deforestation and armed conflict; that social aspect should be studied more deeply (*31*).

As for other emerging infectious diseases, ecologic disruptions could generate changes in the transmission, epidemiology, and distribution of yellow fever in the region (*32*). On the other hand, social determinants play a key role in yellow fever outbreaks, as they do in other neglected tropical diseases. In Colombia, reported cases are correlated with indigenous peoples, agricultural workers, deforested regions, and zones of armed conflict (*2*,*33*).

Since October 2024, Colombia has been experiencing a significant yellow fever outbreak, characterized by sylvatic cycle transmission and a large number of deaths in humans and NHPs in the department of Tolima, compared with the low national vaccination coverage of 64% among persons 1–59 years of age during 1996–2024. In Tolima, vaccination coverage was <50% for the 15–59 age group (*34*). A similar outbreak occurred in Brazil during 2016–2018, which mainly affected unvaccinated persons in areas that were not considered endemic for yellow fever (*35*). That outbreak marked a high number of human and NHP deaths from this virus since 1980 (*35*).

Measures to contain outbreaks of yellow fever include achieving vaccination coverage of >95% in at-risk areas and strengthening entomological and epizootic surveillance, vector control, and risk communication (*36*). Surveillance of epizootics through illness and death rates in NHPs in medium- or high-risk areas is a measure that contributes to the early detection of potential outbreaks before human cases appear (*37*). In addition, Brazil has analyzed the importance of genomic epidemiology in understanding the dynamics and spatial corridor of the yellow fever outbreak in southern Brazil (*38*). Colombia must incorporate genomic surveillance to clarify the circulating lineages of the virus and their relationship with vaccination coverage and eco-epidemiology in different territories. Lessons learned would be applied to other nations in South America also facing similar challenges from ongoing outbreaks of yellow fever (*39*). 

To translate those ideas into practice, we propose a package of complementary strategies that health authorities can adopt during and after the current outbreak. First, sustained genomic surveillance of circulating yellow fever strains should be institutionalized to detect viral lineages, mutations, and transmission pathways in real time. Second, permanent One Health monitoring networks that integrate human, primate, and vector data must be established, enabling early detection of epizootics and vector expansion across ecologic corridors (*40*). Third, vaccination strategies should be adapted to include not only historically high-risk areas but also regions newly exposed to yellow fever because of deforestation, migration, and climate change; coverage should be sustained above the 95% threshold. Finally, those measures should be supported by intersectoral collaboration of public health, environmental, and veterinary authorities to provide a coordinated response that strengthens preparedness and resilience.

## Discussion

The resurgence of yellow fever in Colombia during 2024–2025, particularly in the Magdalena Valley region, underscores the enduring threat of this arboviral disease in South America. Although yellow fever is a vaccine-preventable illness, the outbreak has exposed major gaps in Colombia’s public health infrastructure, especially regarding vaccination coverage, vector and epizootic surveillance, and One Health preparedness. The outbreak through mid-2025, centered in the department of Tolima, represents a significant yellow fever outbreak in Colombia in the 21st Century with 132 confirmed human cases and 68 confirmed infections in NHPs. The crisis reflects both historical patterns and novel ecologic and epidemiologic dynamics shaped by deforestation, habitat disruption, climate change, and sociopolitical factors.

Historically, Colombia has faced recurrent yellow fever outbreaks since colonial times, with notable occurrences in the 19th and 20th Centuries. However, the assumption that some regions, such as Tolima, were at low or negligible risk for yellow fever has proven dangerously misleading. That misclassification hindered preventive measures such as proactive vaccination campaigns, epizootic surveillance, and vector control programs in areas now recognized as vulnerable. The reappearance of yellow fever in Tolima, a region not historically associated with high yellow fever transmission in the 21st Century, reiterates the critical importance of adopting dynamic, data-driven risk stratification models that reflect ecologic changes and disease emergence patterns.

The current outbreak demonstrates the consequences of delayed and reactive public health responses. Although Colombia’s Ministry of Health declared a public health emergency in 2025, the measures came only after widespread transmission among both humans and NHPs. This delayed response highlights a persistent weakness in the integration of One Health approaches, which emphasize early detection through sentinel species monitoring, particularly among NHP populations that share ecologic niches with human communities. The lack of sustained surveillance of illness and deaths in NHPs as well as vector densities is a missed opportunity to anticipate and prevent the current outbreak.

In addition, the intersection of ecologic degradation and yellow fever transmission cannot be ignored. Deforestation, habitat fragmentation, and the displacement of NHPs have brought vectors, wildlife, and humans into closer contact, intensifying the likelihood of spillover events. Those environmental pressures, coupled with the expansion of agricultural frontiers and illicit economies, have drastically altered the eco-epidemiology of yellow fever. Armed conflict further complicates access to vaccination and healthcare in many affected regions, especially in rural and indigenous communities. Those social determinants of health must be addressed as integral components of disease control strategies. We propose an integrated framework that links genomic surveillance, One Health monitoring of NHPs and vectors, and systematic evaluation of socioecologic drivers such as deforestation, land-use change, and armed conflict, thereby offering a robust scientific basis for anticipating, preventing, and more effectively responding to yellow fever outbreaks in Colombia.

The low national yellow fever vaccination coverage—64% among those 1–59 years of age and <50% in Tolima for adults 15–59 years of age—constitutes a major vulnerability. This situation mirrors the Brazil outbreak of 2016–2018, when low immunization in areas previously deemed low-risk enabled widespread transmission. Colombia must urgently reassess its vaccination policies and implement a robust, nationwide campaign targeting not only known endemic areas but also ecologically sensitive regions currently undergoing anthropogenic change.

## Conclusions

Moving forward, Colombia must institutionalize a truly intersectoral One Health approach that integrates ecologic, veterinary, and human health surveillance systems. Strengthening genomic epidemiology capacities is also imperative for tracking yellow fever viral lineages, assessing transmission pathways, and monitoring vaccine escape or mutation events. Those actions, combined with risk communication and community engagement, will enhance outbreak preparedness and foster long-term resilience.

Beyond its human toll, the current outbreak has important economic implications. Many of the affected regions overlap with areas of agricultural production, oil extraction, and mining, sectors that are central to Colombia’s national economy. Interruptions in labor productivity caused by illness or death, combined with restrictions in high-risk zones, can reduce output and generate losses. In addition, tourism, particularly ecotourism in natural parks and forested areas such as Tolima and the Amazon, is susceptible to travel advisories and public perceptions of risk, which could cause declines in both domestic and international visitor numbers. Those effects highlight that yellow fever is not only a health issue but also a threat to economic stability and development. If outbreaks are perceived as undermining income and productivity, particularly in extractive and service industries, political motivation to strengthen vaccination, surveillance, and vector control may increase. Yet, sustained preparedness requires more than reactive measures; it demands continuous allocation of resources from national budgets, complemented by international cooperation and multilateral support, to build resilient One Health infrastructure capable of preventing future crises.

In summary, the current yellow fever outbreak in Colombia is both a public health emergency and a warning. It reflects a failure to learn from historical precedent and to act on mounting ecologic and epidemiologic signals. Colombia, and other South America nations facing similar risks, must prioritize preventive vaccination, entomological and epizootic surveillance, ecologic preservation, and equitable healthcare access. Without decisive action, the cycle of emergence, devastation, and reactive response will persist, putting both human and animal populations at continued risk.

## References

[1] World Health Organization. Yellow fever. 2023 [cited 2025 Nov 22]. https://www.who.int/news-room/fact-sheets/detail/yellow-fever

[2] Ministerio de Salud y Protección Social de Colombia. Guideline for comprehensive clinical care of yellow fever in Colombia [in Spanish]. 2024 [cited 2025 Nov 22]. https://www.minsalud.gov.co/sites/rid/Lists/BibliotecaDigital/RIDE/VS/PP/ET/lineamiento-atencion-clinica-integral-fiebre-amarilla-2024.pdf

[3] Patiño Camargo L. Notes on yellow fever in Colombia [in Spanish]. Rev Fac Med (Caracas). 1937;6:211–81 https://revistas.unal.edu.co/index.php/revfacmed/article/view/23549.

[4] Gast Galvis A. History of yellow fever in Colombia [in Spanish]. Bogotá: Imprenta del Instituto Nacional de Salud; 1982.

[5] Gast Galvis A. Incidence of yellow fever in different areas of Colombia [in Spanish]. Bol Oficina Sanit Panam OSP. 1958;44. 13499620

[6] Boshell Manrique J. Report on wild yellow fever in the Meta region, from July 1934 to December 1936 [in Spanish]. Rev Fac Med (Caracas). 1938;6:407–27 https://revistas.unal.edu.co/index.php/revfacmed/article/view/24090.

[7] Gast Galvis A. Examination results of the first 5,000 human liver samples obtained in Colombia for the study of yellow fever [in Spanish]. Rev Fac Med (Caracas). 1941;10:87–112 https://revistas.unal.edu.co/index.php/revfacmed/article/view/25974.

[8] Corredor Arjona A. Yellow fever in Colombia: a seminal investigation [in Spanish]. Rev Salud Publica (Bogota). 1999;1:137–51.

[9] Instituto Nacional de Salud de Colombia. Study of a yellow fever outbreak in Caquetá, 2005 [in Spanish]. Inf Quinc Epidemiol Nac. 2006;11:329–44.

[10] Segura ÁM, Cardona D, Garzón MO. [Trends in yellow fever mortality in Colombia, 1998-2009] [in Spanish]. Biomedica. 2013;33(Suppl 1):52–62. 24652249

[11] Pan American Health Organization. Reported cases of notifiable diseases in the Americas, 1964 [in Spanish]. 1966 [cited 2025 Nov 22]. https://iris.paho.org/handle/10665.2/47821

[12] Pan American Health Organization. Yellow fever in the Americas. PAHO Epidemiol Bull. 1980 [cited 2025 Nov 22]. https://iris.paho.org/handle/10665.2/46432

[13] Pan American Health Organization. Yellow fever in the Americas, 1981–1985. Bol Epidemiol. 1986;7:1–2. 4052698

[14] Instituto Nacional de Salud de Colombia. Yellow fever in Colombia, 2000–2019 [in Spanish]. Boletín Epidemiológico Semanal. Report no. 4. 2020 [cited 2025 Nov 22]. https://www.ins.gov.co/buscador-eventos/BoletinEpidemiologico/2020_Boletin_epidemiologico_semana_4.pdf

[15] Instituto Nacional de Salud de Colombia. Event report: yellow fever [in Spanish]. 2025 Mar 22 [cited 2025 Nov 22] https://www.ins.gov.co/buscador-eventos/Informesdeevento/FIEBRE%20AMARILLA%20PE%20III%202025.pdf

[16] Sanchez-Rojas IC, Solarte-Jimenez CL, Chamorro-Velazco EC, Diaz-Llerena GE, Arevalo CD, Cuasquer-Posos OL, et al. Yellow fever in Putumayo, Colombia, 2024. New Microbes New Infect. 2025;64:101572. 40129853 10.1016/j.nmni.2025.101572 PMC11932672

[17] Pan American Health Organization. National profile of yellow fever in Colombia [in Spanish]. 2022 [cited 2025 Nov 22]. https://iris.paho.org/bitstream/handle/10665.2/56910/OPSFPLIM0039_spa.pdf?sequence=1&isAllowed=y

[18] Instituto Nacional de Salud de Colombia. SIVICAP Evaluation of chemical and microbiological risk in drinking water in Colombia, 2023 [in Spanish]. Boletín Epidemiológico Semanal. Report no. 43. 2024 [cited 2025 Nov 22].https://www.ins.gov.co/buscador-eventos/BoletinEpidemiologico/2024_Boletin_epidemiologico_semana_43.pdf

[19] Instituto Nacional de Salud de Colombia. Measles and rubella: epidemiological behavior of integrated surveillance [in Spanish]. Boletín Epidemiológico Semanal. Report no. 37. 2025 [cited 2025 Nov 22]. https://www.ins.gov.co/buscador-eventos/BoletinEpidemiologico/2025_Boletin_epidemiologico_semana_37.pdf

[20] Ministerio de Salud y Protección Social de Colombia. Resolution 691 of 2025. Through which a health emergency is declared throughout the national territory due to the outbreak caused by the yellow fever virus, and measures are adopted for its prevention and control [in Spanish]. 2025 [cited 2025 Nov 22]. https://www.minsalud.gov.co/Normatividad_Nuevo/Resolucion%20No%20691%20de%202025.pdf

[21] Perez LJ, Perez-Restrepo LS, Ciuoderis K, Usuga J, Moreno I, Vargas V, et al. Emergence, persistence, and positive selection of yellow fever virus in Colombia. Front Microbiol. 2025;16:1548556. 40260085 10.3389/fmicb.2025.1548556 PMC12009951

[22] Piedrahita-Cortés J, Soler-Tovar D. Geographical distribution of the red howler monkey (*Alouatta seniculus*) and yellow fever in Colombia. Biomedica. 2016;36:116–24. 27622801 10.7705/biomedica.v36i0.2929

[23] Hernández-Galvis J, Pizarro AB, Cuestas JA, Castañeda-Cardona C, Rosselli D. Yellow fever in Colombia: from public calamity to neglected disease. Acta Méd Peruana. 2018;35:55–9.

[24] Instituto Nacional de Salud de Colombia. Yellow fever [in Spanish]. Boletín Epidemiológico Semanal. Report no. 4. 2024 [cited 2025 Nov 22]. https://www.ins.gov.co/buscador-eventos/BoletinEpidemiologico/2024_Bolet%C3%ADn_epidemiologico_semana_4.pdf

[25] Nederlof RA, Virgilio T, Stemkens HJJ, da Silva LCCP, Montagna DR, Abdussamad AM, et al. Yellow fever in non-human primates: a veterinary guide from a One Health perspective. Vet Sci. 2025;12:339. 40284841 10.3390/vetsci12040339 PMC12031500

[26] Berthet M, Mesbahi G, Duvot G, Zuberbühler K, Cäsar C, Bicca-Marques JC. Dramatic decline in a titi monkey population after the 2016-2018 sylvatic yellow fever outbreak in Brazil. Am J Primatol. 2021;83:e23335. 34609763 10.1002/ajp.23335

[27] Organización Panamericana de la Salud. Epidemiological update on yellow fever in the Americas Region [in Spanish]. 2025 [cited 2025 Nov 22]. https://www.paho.org/sites/default/files/2025-04/2025-abril-24-phe-actualizacion-epidemiologica-fiebre-amarilla-final.pdf

[28] Aliaga-Samanez A, Real R, Segura M, Marfil-Daza C, Olivero J. Yellow fever surveillance suggests zoonotic and anthroponotic emergent potential. Commun Biol. 2022;5:530. 35654842 10.1038/s42003-022-03492-9 PMC9163115

[29] Cruz-Rodríguez C, Noguera-Urbano E, Olaya-Rodríguez MH, Henao-Díaz LF, Guzmán-Caro DC, Ochoa-Quintero JM, et al. Primates and deforestation in Colombia. Alexander von Humboldt Biological Resources Research Institute. 2019 [cited 2025 Nov 22]. https://reporte.humboldt.org.co/biodiversidad/2019/cap2/204

[30] Henao Diaz F, Stevenson P, Carretero-Pinzón X, Castillo-Ayala C, Chacón Pacheco J, Defler T, et al. Biodiversity atlas of Colombia. Primates. Alexander von Humboldt Biological Resources Research Institute. 2020 [cited 2025 Nov 22]. https://biomodelos.humboldt.org.co/atlas/Primates.pdf

[31] García Botero R; Instituto Colombo-Alemán para la Paz. Deforestation and armed conflict in Colombia: considerations and recommendations. San Francisco: Academia; 2024.

[32] McMichael AJ. Environmental and social influences on emerging infectious diseases: past, present and future. Philos Trans R Soc Lond B Biol Sci. 2004;359:1049–58. 15306389 10.1098/rstb.2004.1480 PMC1693387

[33] Tuells J, Henao-Martínez AF, Franco-Paredes C. Yellow fever: a perennial threat. Arch Med Res. 2022;53:649–57. 10.1016/j.arcmed.2022.10.005 36404585

[34] Ministerio de Salud y Protección Social de Colombia. Technical and operational guidelines for the national yellow fever vaccination campaign in the susceptible population aged 15 to 18 years in Colombia. 2024 [cited 2025 Nov 22]. https://www.minsalud.gov.co/sites/rid/Lists/BibliotecaDigital/RIDE/VS/PP/PAI/lineamientos-tecnicos-operativos-jonada-nacional-vacunacion-fiebre-amarilla-15-18-anos.pdf

[35] Santos JD, Rocha KLS, Amaral CD, Dutra AGS, Figueiredo PO, Rocha-Vieira E, et al. The yellow fever outbreak in Brazil (2016–2018): how a low vaccination coverage can contribute to emerging disease outbreaks. Microorganisms. 2025;13:1287. 40572175 10.3390/microorganisms13061287 PMC12195118

[36] Pan American Health Organization. Epidemiological alert yellow fever in the Americas Region—31 May 2025. 2025 [cited 2025 Nov 22]. https://www.paho.org/en/documents/epidemiological-alert-yellow-fever-americas-region-31-may-2025

[37] Bonilla-Aldana DK, Bonilla-Aldana JL, Castellanos JE, Rodriguez-Morales AJ. Importance of epizootic surveillance in the epidemiology of yellow fever in South America. Curr Trop Med Rep. 2025;12:1–9. 10.1007/s40475-025-00349-z

[38] Giovanetti M, Pinotti F, Zanluca C, Fonseca V, Nakase T, Koishi AC, et al. Genomic epidemiology unveils the dynamics and spatial corridor behind the yellow fever virus outbreak in southern Brazil. Sci Adv. 2023;9:eadg9204. 10.1126/sciadv.adg9204 PMC10854437 37656782

[39] Rodriguez-Morales AJ, Alhazmi AH, Katime A, Hameed AA, Morales A, Lepetic AC, et al. SLAMVI, ESGITM, EVASG, ALEIMC, GEPI-SEIMC, SEMEVI, and CMTZMV-ACIN. Yellow fever in South America—a plea for action and call for prevention also in travelers from SLAMVI, ESGITM, EVASG, ALEIMC, GEPI-SEIMC, SEMEVI, and CMTZMV-ACIN. Travel Med Infect Dis. 2025;67:102871. 10.1016/j.tmaid.2025.102871 40582475

[40] Sanchez-Rojas IC, Bonilla-Aldana DK, Solarte-Jimenez CL, Bonilla-Aldana JL, Belisario-Tovar M, Ortega-Gómez S, et al. Fatal yellow fever among captive non-human primates in southern Colombia, 2025. Front Vet Sci. 2025;12:1655474. 10.3389/fvets.2025.1655474 40919041 PMC12409288

